# Tumor heterogeneity on contrast-enhanced computed tomography outperforms attenuation ratio in differentiating renal cell carcinoma subtypes: a pathologic correlation study

**DOI:** 10.11604/pamj.2026.53.104.50865

**Published:** 2026-03-01

**Authors:** Anastasia Dewi Elita Araminta, Muhammad Hidayat Surya Atmaja, Widiana Ferriastuti

**Affiliations:** 1 Department of Radiology, Radiology Specialist Education Program, Faculty of Medicine, Airlangga University, Dr. Soetomo General Academic Hospital, Surabaya, Indonesia,; 2 Department of Radiology, Faculty of Medicine, Airlangga University, Dr. Soetomo General Academic Hospital, Surabaya, Indonesia

**Keywords:** Attenuation ratio, CT scan, heterogeneity, histopathology, malignant renal mass, renal cell carcinoma

## Abstract

**Introduction:**

malignant renal masses are frequently identified on computed tomography (CT) in clinical practice; however, noninvasive differentiation between renal mass subtypes, including renal cell carcinoma (RCC) and non-RCC lesions, remains challenging.

**Methods:**

this cross-sectional study included patients undergoing nephrectomy who had preoperative CT imaging and histopathologic evaluation. The attenuation ratio was defined as the ratio of Hounsfield Unit (HU) values between the tumor and the renal cortex. Tumor heterogeneity was independently assessed on a 5-point Likert scale during the corticomedullary phase.

**Results:**

there was no significant difference in attenuation ratio between the RCC and non-RCC groups (p>0.05), whereas the heterogeneity score of renal masses on CT scans was significantly higher in RCC than in non-RCC masses (P<0.05), reflecting the tumor's structural complexity and aggressiveness.

**Conclusion:**

our findings indicate that mass heterogeneity may outperform the attenuation ratio as a diagnostic and prognostic indicator in distinguishing malignant renal mass subtypes. Assessment of heterogeneity on preoperative CT thus holds promise as a valuable radiologic biomarker and may contribute to optimized treatment planning. Larger-scale studies are needed to further substantiate these results.

## Introduction

Renal masses are frequently identified as incidental findings during diagnostic imaging in clinical practice [1]. These lesions may be solid or cystic and can be classified as benign or malignant [2]. Radiological imaging is essential for the detection, characterization, and management of renal masses. While percutaneous renal biopsy can provide a histopathological diagnosis, it is infrequently the first step in diagnosis, allowing for prompt intervention through observation, ablation, or surgery based on the lesion's characteristics [3]. Multiphasic computed tomography (CT) scans offer critical insights through various contrast phases, including non-contrast, corticomedullary, nephrographic, and excretory. These phases are instrumental for assessing tumor vascularization patterns and identifying necrosis, calcification, and fatty components, all of which are related to tumor subtype and malignancy. This information can aid in predicting tumor biological behavior and correlating with patient outcomes [4-6]. The Hounsfield Unit (HU) values obtained from CT scans provide a quantitative means to evaluate tissue density, enabling non-invasive assessment of tumor grade [7]. CT scans hold significant promise for developing predictive algorithms, as extensive research links CT findings with various renal mass subtypes and predicts favorable outcomes [8]. Renal masses are predominantly assessed with CT rather than MRI, owing to shorter scan times, lower costs, and greater accessibility [9]. To enhance the prediction of clear cell renal cell carcinoma (ccRCC), two critical CT scan parameters can be employed: the degree of corticomedullary enhancement (ranging from high to low) and the heterogeneity score (assessed on a Likert scale).

This algorithm yields predictive value for ccRCC likelihood, with higher scores correlating with a higher malignancy risk. Previous research found that these parameters exhibited significant diagnostic accuracy in distinguishing ccRCC from other subtypes in small renal lesions. The application of the attenuation ratio, alongside the heterogeneity score, not only has diagnostic significance but also influences clinical outcomes. A higher score may prompt earlier surgical intervention, whereas a lower score could lead to active surveillance, minimizing the risk of overtreatment. Consequently, integrating the attenuation ratio and heterogeneity score into the initial assessment of small renal masses could enhance diagnostic precision, facilitate personalized treatment, and optimize healthcare resource utilization [10,11]. A study by Al Nasibi *et al*. [12] utilized a CT scan algorithm to predict clear-cell renal cell carcinoma (cc-RCC) in solid renal masses measuring ≤ 4 cm, reporting a ccRCC rate of 53%. Their findings indicated that a higher mass-to-cortex attenuation ratio during the corticomedullary phase and increased heterogeneity scores were associated with cc-RCC compared to other diagnoses. Following this, Lemieux *et al*. [13] applied the same CT algorithm to assess clinically solid, small, and localized renal masses for cc-RCC. Unlike Al Nasibi *et al*. study [12] which had a more limited sample, Lemieux's analysis involved a diverse group of patients across multiple institutions and a greater number of readers with varying levels of expertise. The results demonstrated that CT scoring achieved moderate area under the curve (AUC) and positive predictive value (PPV), along with a high negative predictive value (NPV) for diagnosing cc-RCC [13]. Based on the previous discussion, this study aims to analyze attenuation ratios and heterogeneity in CT scans of malignant renal masses relative to histopathological results in post-nephrectomy patients, to contribute to the development of reliable radiological biomarkers for tumor grade prediction.

## Methods

**Study design:** this study used an analytical-observational, cross-sectional research design. The study population was obtained from retrospective data collected at Dr. Soetomo Academic Hospital, Surabaya (Indonesia), from January 2020 to May 2025.

**Study setting and population:** the study population comprised all nephrectomy patients documented in the medical records of Dr. Soetomo General Academic Hospital, Surabaya city (Indonesia), between January 2020 and May 2025. Consecutive sampling was used to select participants. The inclusion criteria consisted of patients aged 18 years or older with a diagnosis of a malignant renal mass, a preoperative abdominal CT scan, nephrectomy (partial or radical), accessible medical records, and post-nephrectomy histopathology data confirming a malignant renal mass. Samples were excluded if they had a history of neo-adjuvant therapy, inadequate CT scan quality, and post-nephrectomy histopathology data showing a benign renal mass.

**Variable of study:** the primary dependent variable in this study was electronic medical record data on the histopathological classification of renal mass tissue following nephrectomy. The primary predictor variables were the attenuation ratio and heterogeneity score.

**Data collection and measurement:** this study involved patients with malignant renal masses who underwent contrast-enhanced abdominal CT scans and subsequent nephrectomy. Initially, we identified subjects using predefined inclusion criteria to ensure only relevant patients were enrolled in the study. The final sample was divided into two groups: RCC and non-RCC. We then assessed the attenuation ratios and heterogeneity scores in both groups. The histopathological renal mass tissue was obtained from nephrectomy. Tissue samples were stained with hematoxylin and eosin, with or without immunohistochemistry, and evaluated microscopically by the designated pathologist. The resulting data were nominal. The renal cell carcinoma (RCC) cohort was categorized into two groups: low-grade (Grade I-II) and high-grade (Grade III-IV). The non-RCC cohort was subdivided into three categories: transitional cell carcinoma (TCC), non-Hodgkin lymphoma (NHL), and metastatic disease. The attenuation ratio and heterogeneity score of renal tissue derived from a 128-slice preoperative CT scan (Philips, Model No. 989000086371, Serial No. 145872, Tube MRC 880, Netherlands). Scans were performed with or without 0.5 mmol/ml of Gadoteric acid contrast (Dotarem, Guerbet LLC, Princeton, USA). Two physicians, under the supervision of a consultant radiologist who was unaware of the histopathology outcomes, independently reviewed the abdominal CT scans.

The density values of the renal lesion relative to the ipsilateral normal renal cortex determined the corticomedullary phase attenuation ratio for a renal mass. During this phase, the attenuation ratio was calculated by dividing the mass attenuation by the attenuation of the renal cortex. The resulting value was classified as low (<0.40), moderate (0.40-0.75), or high (>0.75). Regions of interest (ROIs) are measured at three points within the most hyperattenuating area of mass, each with a diameter of 2 cm, and the average of these measurements was taken. ROIs for the renal cortex were positioned ipsilateral to the mass in the corticomedullary phase using axial slices, ensuring the necrotic areas of the mass were avoided as much as possible. Heterogeneity of the renal mass was evaluated using a modified Likert scale based on corticomedullary CT scans. The assessment results were categorized into five levels: completely homogeneous (the entire area appears homogeneous), mostly homogeneous (a hyperdense area is observed at the mass periphery), mixed homogeneous and heterogeneous (a heterogeneous area appears more uniform along the medial border, with increased homogeneity at the edges), mostly heterogeneous (a predominantly heterogeneous area with a small homogeneous section), and completely heterogeneous (the entire area is heterogeneous).

**Sample size:** the sample size was determined based on predictions of sensitivity and specificity of area under curve (AUC). The parameters utilized included an alpha level of 0.05, an AUC of 0.7, and an allowable estimation error of 0.2. This analysis resulted in a minimum sample size of 26 participants.

**Data analysis:** all statistical analyses were conducted using SPSS version 26.0 (IBM, USA). The initial phase focused on evaluating the baseline demographic and clinical characteristics of the cohort. All data are expressed in frequencies and percentages according to variables categories. To assess the association between attenuation ratio and heterogeneity with histopathological outcomes of RCC and non-RCC, simple logistic regression was employed. For more detailed histopathological results, a subgroup analysis was performed using Fisher's exact test. A significance level of α = 0.05 was utilized for all inferential analyses, with results expressed as odds ratios (OR) and corresponding 95% confidence intervals where applicable. This study did not conduct subgroup analysis, handling missing data, and sensitivity analysis.

**Ethical consideration:** the procedures received ethical approval from the Health Ethical Committee of Airlangga University (Letter No. 2054/LOE/301.4.2/VII/2025 Protocol No. 3777/118/4/VII/2025). Confidentiality was maintained, and data access was restricted to authorized investigators.

## Results

The study encompassed data collected from January 2020 to May 2025, involving 1,112 patients diagnosed with a malignant renal mass according to ICD-10 criteria. Following screening based on abdominal CT scan results, 134 patients were deemed eligible for inclusion. However, 103 patients were excluded due to the absence of histopathology data, diagnoses of benign renal masses, or prior neo-adjuvant therapy. Consequently, a total of 31 patients were included in the statistical analysis

**Baseline characteristics:** Table 1 presents the baseline characteristics of the study participants. Most subjects were female and aged 51-60 years.

**Table 1:** baseline characteristics of research subjects recruited from Dr. Soetomo General Academic Hospital, Surabaya (Indonesia), between January 2020 and May 2025 (N=31)

Characteristics	Frequency (%)		
	RCC (n=23)	Non-RCC (n=8)	Total (N=31)
**Age (years)**			
18-30	3	0	3 (9.6%)
31-40	3	1	4 (12.9%)
41-50	4	1	5 (16.1%)
51-60	8	2	10 (32.2%)
61-70	6	3	8 (25.8%)
71-80	0	1	1 (3.2%)
**Gender**			
Man	7	5	12 (38.7%)
Woman	16	3	19 (61.3%)

RCC: renal cell carcinoma

**Predictive value of attenuation ratio and heterogeneity score toward renal cell carcinoma:** Table 2 presents an analysis of the predictive value of the attenuation ratio and the heterogeneity score concerning RCC status. The attenuation ratio did not demonstrate a significant predictive value for RCC (OR 2.572, 95% CI: 0.594-11.126). In contrast, the heterogeneity score exhibited a significant predictive value for RCC (OR 3.955, 95% CI: 1.013-15.451).

**Table 2:** predictive value of attenuation ratio and heterogeneity score for RCC in Dr. Soetomo General Academic Hospital, Surabaya (Indonesia), from January 2020 to May 2025

Predictors	Histopathology results (n, %)		P-value	OR (95% CI)
	RCC (n=23)	Non-RCC (n=8)		
**Attenuation ratio**				
Low (<0.4)	0 (0.0%)	1 (2.5%)	0.206	2.572 (0.594-11.126)
Moderate (0.4-0.75)	5 (21.7%)	2 (25.0%)		
High (>0.75)	18 (78.3%)	5 (62.5%)		
**Heterogeneity score**				
Completely homogenous	0 (0.0%)	0 (0.0%)	0.048*	3.955 (1.013-15.451)
Mostly homogenous	0 (0.0%)	2 (25%.0)		
Mixed	0 (0.0%)	1 (12.5%)		
Mostly heterogeneous	2 (8.7%)	0 (0.0%)		
Completely heterogeneous	21 (91.3%)	5 (62.5%)		

RCC: Renal cell carcinoma, (*): significant p value (p<0.05)

**Association of attenuation ratio and heterogeneity score with histopathological assessment of renal cell carcinoma:** Table 3 presents the analysis of the relationships among the attenuation ratio, heterogeneity score, and RCC histopathology grading. The findings indicate that most RCC cases exhibited a high attenuation ratio, irrespective of the histopathological grade. Additionally, nearly all RCC cases fell into the completely heterogeneous category (the highest score), with 88.9% for low-grade RCC and 92.9% for high-grade RCC. No significant association was found between the attenuation ratio, the heterogeneity score, and RCC histopathology grading. Table 4 presents an analysis of the relationship between the attenuation ratio and heterogeneity score across RCC tumor subtypes. A significant majority of RCC cases exhibited a high attenuation ratio (>0.75), with histopathological assessments primarily identifying the clear cell RCC subtype. Almost all RCC cases fell into the completely heterogeneous category, with the highest heterogeneity score, and clear cell RCC was the predominant subtype. While the RCC tumor subtype showed a significant association with the heterogeneity score, it did not correlate with the attenuation ratio. Table 5 presents the analysis of the relationship between the attenuation ratio and heterogeneity score in relation to the size of RCC tumor masses. The largest sample population was observed in masses greater than 10 cm, characterized by a high attenuation ratio. Additionally, the highest sample population based on the heterogeneity score was also found in masses over 10 cm, which exhibited a completely heterogeneous heterogeneity score. However, this study found no significant association between tumor mass size and either the attenuation ratio or the heterogeneity score of RCC tumors.

**Table 3:** relationship between attenuation ratio and heterogeneity score with histopathological grading of RCC in Dr. Soetomo General Academic Hospital, Surabaya (Indonesia), from January 2020 to May 2025

Parameters	Histopathological grading (n, %)		P-value
	Low (n=9)	High (n=14)	
**Attenuation ratio**			
Low (<0.4)	0 (0.0%)	0 (0.0%)	1.000
Moderate (0.4-0.75)	2 (22.2%)	3 (21.4%)	
High (>0.75)	7 (77.8%)	11 (78.6%)	
**Heterogeneity score**			
Completely homogenous	0 (0.0%)	0 (0.0%)	1.000
Mostly homogenous	0 (0.0%)	0 (0.0%)	
Mixed	0 (0.0%)	0 (0.0%)	
Mostly heterogeneous	1 (11.1%)	1 (7.1%)	
Completely heterogeneous	8 (88.9%)	13 (92.9%)	

RCC: Renal Cell Carcinoma; Fisher exact test

**Table 4:** relationship between attenuation ratio and heterogeneity score with RCC tumor subtype in Dr. Soetomo General Academic Hospital, Surabaya (Indonesia), from January 2020 to May 2025

Parameter	Tumor Subtype (n, %)				P-value
	cc-RCC (n=17)	p-RCC (n=3)	ch-RCC (n=2)	tub-RCC (n=1)	
**Attenuation ratio**					
Low (<0.4)	0 (0.0%)	0 (0.0%)	0 (0.0%)	0 (0.0%)	0.341
Moderate (0.4-0.75)	4 (23.5%)	0 (0.0%)	0 (0.0%)	1 (100%)	
High (>0.75)	13 (76.5%)	3 (100%)	2 (100%)	0 (0.0%)	
**Heterogeneity score**					
Completely homogenous	0 (0.0%)	0 (0.0%)	0 (0.0%)	0 (0.0%)	0.036*
Mostly homogenous	0 (0.0%)	0 (0.0%)	0 (0.0%)	0 (0.0%)	
Mixed	0 (0.0%)	0 (0.0%)	0 (0.0%)	0 (0.0%)	
Mostly heterogeneous	0 (0.0%)	1 (33.3%)	0 (0.0%)	1 (100%)	
Completely heterogeneous	17 (100%)	2 (66.7%)	2 (100%)	0	

cc: clear cell, ch: chromophobe, p: papillary, RCC: renal cell carcinoma, tub: tubulomucinous Fisher exact test, *significant at P<0.05

**Table 5:** relationship between attenuation ratio and heterogeneity score with RCC tumor mass size on CT scan in Dr. Soetomo General Academic Hospital, Surabaya (Indonesia), from January 2020 to May 2025

Parameters	Mass Size on CT-Scan (n, %)				P-value
	< 4 cm (n=1)	4-7 cm (n=6)	8-10 cm (n=3)	> 10 cm (n=13)	
**Attenuation ratio**					
Low (<0.4)	0 (0.0%)	0 (0.0%)	0 (0.0%)	0 (0.0%)	1.000
Moderate (0.4-0.75)	0 (0.0%)	1 (16.7%)	1 (33.3%)	3 (23.1%)	
High (>0.75)	1 (100%)	5 (83.3%)	2 (66.7%)	10 (76.9%)	
**Heterogeneity score**					
Completely homogenous	0 (0.0%)	0 (0.0%)	0 (0.0%)	0 (0.0%)	1.000
Mostly homogenous	0 (0.0%)	0 (0.0%)	0 (0.0%)	0 (0.0%)	
Mixed	0 (0.0%)	0 (0.0%)	0 (0.0%)	0 (0.0%)	
Mostly heterogeneous	0 (0.0%)	1 (16.7%)	0 (0.0%)	1 (7.7%)	
Completely heterogeneous	1 (100%)	5 (83.3%)	2 (100%)	13 (92.3%)	

RCC: Renal cell carcinoma

**Association of attenuation ratio and heterogeneity score with non-renal cell carcinoma histopathology outcomes:** Table 6 presents an analysis of the relationship between the attenuation ratio and heterogeneity score across non-RCC tumor subtypes. The majority of samples exhibited a high attenuation ratio (>0.75) within TCC histopathology. Most patients with non-RCC histopathology fell into the completely heterogeneous category, with TCC being the predominant tumor subtype. However, no significant associations were observed between tumor subtype, attenuation ratio, and heterogeneity score for non-RCC tumors in this study.

**Table 6:** relationship between attenuation ratio and heterogeneity score with tumor subtype of non-RCC cases in Dr. Soetomo General Academic Hospital, Surabaya (Indonesia), from January 2020 to May 2025

Parameters	Tumor subtype (n, %)			P- value
	TCC (n=6)	NHL (n=1)	Metastasis (n=1)	
**Attenuation ratio**				
Low (<0.4)	1 (16.7%)	0 (0.0%)	0 (0.0%)	0.643
Moderate (0.4-0.75)	0 (0.0%)	1 (100%)	0 (0.0%)	
High (>0.75)	5 (83.3%)	0 (0.0%)	1 (100%)	
**Heterogeneity score**				
Completely homogenous	0 (0.0%)	0 (0.0%)	0 (0.0%)	0.286
**Mostly homogenous**	2 (33.3%)	0 (0.0%)	0 (0.0%)	
Mixed	0 (0.0%)	1 (100%)	0 (0.0%)	
Mostly heterogeneous	0 (0.0%)	0 (0.0%)	0 (0.0%)	
Completely heterogeneous	4 (66.7%)	0 (0.0%)	1 (100%)	

## Discussion

The CT scan attenuation ratio data and histopathology results in the RCC group in this study showed that most of the sample population had a high attenuation ratio, with histopathology indicating high-grade RCC. This finding is consistent with the radiological characteristics of RCC, especially the clear cell RCC subtype, which generally shows increased density after contrast administration due to its rich vascularization and hypervascular tumor tissue [6]. This can be explained by the biological nature of RCC, which tends to be angiogenic and associated with VHL gene mutations that trigger the expression of vascular endothelial growth factor (VEGF), thereby increasing perfusion and contrast accumulation on CT imaging [14]. Research by Walker *et al*. [15] also reported that higher attenuation values on multiphase contrast CT were closely associated with the diagnosis of RCC, especially in the clear cell subtype, compared to non-RCC tumors, which showed relatively lower attenuation and different enhancement patterns. Meanwhile, Chen *et al*. [16] noted that quantitative CT density measurement can serve as a supporting parameter for differentiating RCC from other renal neoplasms. However, it cannot be used as the sole diagnostic criterion. The analysis of CT scan attenuation ratios relative to tumor size indicates that the largest cohort consists of tumors over 10 cm in size with elevated attenuation ratios. Research consistently demonstrates a correlation between renal mass size and increased attenuation on CT imaging. Larger masses typically exhibit higher attenuation densities, attributed to variations in tumor composition, such as necrosis, hemorrhage, and dense tissue with intricate vascularization. For instance, Zhang *et al*. [5] reported that tumor size correlates with the extent of enhancement during the contrast phase, with larger lesions displaying a more heterogeneous enhancement pattern. Similarly, Young *et al*. [17] found that renal masses exceeding 4 cm are more likely to exhibit significant attenuation variation, particularly in RCC subtypes.

Additionally, Galia *et al*. [18] noted that larger tumors tend to exhibit more complex radiological features, which can influence measured attenuation values. The CT scan attenuation ratio data, in conjunction with histopathological findings, revealed that most samples in the non-RCC group exhibited a high attenuation ratio alongside TCC-type histopathology. This observation is noteworthy, as TCC of the kidney or renal pelvis typically demonstrates moderate to high enhancement after contrast administration, which reflects its histological characteristics, including a relatively dense stroma and moderate vascularization [19]. Supporting evidence is found in several prior studies. For instance, Walker *et al*. [15] noted that TCC generally shows higher attenuation values than benign lesions, such as complex cysts or oncocytomas. However, these values are not as elevated as those seen in clear cell RCC. A homogeneous enhancement pattern with increased attenuation during the corticomedullary or nephrographic phase has been identified as a distinguishing feature that helps differentiate TCC from other renal tumors [20]. Furthermore, Kim *et al*. [21] demonstrated that CT attenuation ratios enhance diagnostic accuracy when distinguishing TCC from non-epithelial renal neoplasms. A high attenuation ratio in TCC is indicative of a more cellular tumor composition and minimal necrotic areas, resulting in a relatively uniform appearance on contrast-enhanced CT scans. A recent retrospective study by Bata *et al*. [22] found that the attenuation ratio of tumor to normal renal parenchyma during the nephrographic and corticomedullary phases is significantly higher in RCC than in TCC. However, TCC can exhibit a high attenuation ratio when presenting homogeneous features, complicating differentiation based solely on this parameter. Additionally, Kulkarni *et al*. [23] demonstrated that in multiphasic CT protocols, TCC frequently shows early arterial-phase enhancement and contrast enhancement that may resemble vascular lesions, such as RCC. Thus, a high attenuation ratio is not a definitive indicator of clear cell renal malignancy [24]. These findings highlight that while a high attenuation ratio may suggest TCC, it lacks specificity without considering the lesion's anatomical location (renal pelvis versus parenchyma) and the complete enhancement pattern. Therefore, it is essential to integrate the attenuation ratio with morphological characteristics (including lesion shape and distribution) and clinical context for a more accurate diagnosis. This study compared histopathology-based attenuation ratios between the RCC and non-RCC groups. The findings revealed no significant difference in attenuation ratios between the two groups. This outcome aligns with prior research that identified the limitations of using attenuation ratios in CT scans for distinguishing renal tumor subtypes. While CT attenuation values can offer diagnostic insights, there exists considerable overlap between RCC and non-RCC, which affects the reliability of this measure alone. Similarly, Lee *et al*. [25] reported that absolute Hounsfield unit (HU) values and phase-contrast attenuation ratios were inadequate for differentiating all RCC subtypes or for distinguishing them from oncocytomas.

The results of this analysis indicate no significant difference in the attenuation ratios between the RCC and non-RCC groups. One contributing factor may be tumor size. Previous studies have shown that small renal tumors (<4 cm) often exhibit atypical enhancement patterns, resulting in overlapping attenuation ratios between RCC and non-RCC. In contrast, larger tumors (>4-5 cm) typically demonstrate more heterogeneous enhancement in RCC due to necrosis, bleeding, or calcification, whereas non-RCC tumors remain hypodense with minimal enhancement. Despite visual differences across imaging, the numerical attenuation ratios frequently overlap, especially when measured using the average ROI method [5,25]. Additionally, the increasing internal heterogeneity of RCC with tumor growth can obscure differences in attenuation values relative to non-RCC. The selection of ROI location is critical; measuring the viable tumor area may highlight more distinct differences, whereas averaging across the entire mass diminishes sensitivity. Non-RCCs, which commonly arise from the pelvicalyceal system, are often detected at smaller sizes with low enhancement levels. The relationship between CT scan attenuation ratios and microvascularization is complex, influenced by factors such as blood vessel heterogeneity, necrosis, bleeding, and contrast distribution within the lesion [5]. Angiogenesis in RCC exhibits fundamental similarities to that in non-RCC renal tumors, including TCC, NHL, and metastases. All these tumor types require the formation of new blood vessels to sustain growth and dissemination, with key mediators like VEGF playing an essential role across all solid renal neoplasms [26]. In this study, the characteristics of the samples: i) predominantly exceeding 10 cm in size and varying in mass composition; ii) further complicated the distinction in attenuation ratios between the RCC and non-RCC groups. A study conducted by Sasaguri *et al*. [27] revealed that while the attenuation ratios between RCC and oncocytoma were not significantly different, oncocytoma exhibited a more pronounced increase in specific imaging signs; iii) particularly during the nephrographic and excretory phases; iv) compared to RCC. These findings reinforce the notion that the attenuation ratio alone is insufficient for distinguishing RCC from non-RCC renal tumors and should be evaluated alongside other imaging characteristics.

In this study, most malignant renal mass samples were classified as completely heterogeneous, exhibiting the highest scores across both low- and high-grade tumors. Notably, clear cell renal cell carcinoma (ccRCC) was the predominant subtype and was also categorized as completely heterogeneous. This observation aligns with the biological characteristics of RCC, particularly ccRCC, which is known for its hypervascularity and the presence of diverse intra-tumor components, including prominent enhancement along with features such as necrosis, bleeding, calcification, and stromal changes. These elements contribute to significant variations in attenuation among pixels and voxels, resulting in a distinctly heterogeneous CT appearance. This image heterogeneity is conceptually indicative of biological heterogeneity, reflecting heterogenous angiogenesis, hypoxia, and necrosis caused by imbalance process between oxygen supply and demand, as well as extracellular matrix remodeling. Such variability has been consistently linked to higher malignancy grades (WHO/ISUP grade) and various markers of tumor aggressiveness. CT scans indicated that the largest sample population consisted of masses exceeding 10 cm, all exhibiting a completely heterogeneous heterogeneity score. This observation aligns with existing literature, which indicates that tumor heterogeneity on CT scans becomes more pronounced in larger tumors (>10 cm). As tumors grow, they often develop areas of central necrosis, bleeding, and cystic degeneration, leading to images with considerable density and attenuation variation across tumor regions [28]. Additionally, other research has demonstrated that in larger renal tumors, changes in microvascularization and hypoxia-induced cell death contribute to increasingly non-uniform imaging patterns between the lesion's center and periphery [29].

The results of the CT heterogeneity score analysis in the non-RCC group showed that most cases, particularly TCC and renal NHL, were in the most heterogeneous category (highest score). This finding is interesting because radiological image heterogeneity is not only a characteristic of RCC but can also occur in non-RCC renal tumors with different biological mechanisms. In TCC, heterogeneous images are often due to infiltrative growth patterns, focal necrosis, and involvement of the pelvicalyceal system, which lead to density variations. TCC can exhibit heterogeneity due to a combination of solid and necrotic components, leading to variations in CT attenuation. Furthermore, Lee *et al*. [25] emphasized that although TCC is generally hypovascular compared to RCC, the degree of heterogeneity can increase in large lesions or those with peripelvic invasion. Conversely, renal NHL is typically characterized by a more homogeneous and hypodense appearance on CT scans. However, in certain cases, particularly those involving variations in immune response, necrosis, or multifocal involvement, increased heterogeneity may be observed. Liu *et al*. [11] highlighted that heterogeneity in lymphoma may arise from necrotic processes due to rapid tumor growth. Overall, the findings from this study align with existing literature, indicating that high heterogeneity scores are not exclusive to RCC but may also occur in TCC and renal lymphoma under specific circumstances. Therefore, while heterogeneity can help rule out benign lesions, a comprehensive diagnostic interpretation must still account for the multiphase enhancement pattern, anatomical distribution, and clinical features to distinguish RCC from non-RCC conditions. This study revealed a statistically significant difference in heterogeneity distribution between RCC and non-RCC cases. Lesions exhibiting heterogeneous characteristics were predominantly found in the RCC group, while those with homogeneous features were more prevalent in the non-RCC group. These results suggest that radiological image heterogeneity may serve as a crucial parameter for distinguishing RCC from non-RCC renal tumors, potentially enhancing diagnostic accuracy in initial imaging assessments. The supporting literature indicates that radiological heterogeneity is often associated with histopathological features such as necrosis, bleeding, and varying levels of intratumoral vascularization, which are infrequently observed in benign tumors [30]. Additionally, studies using CT-based radiomics have demonstrated that analyzing texture and heterogeneity can help differentiate renal tumor subtypes and predict their biological behavior [31].

In this study, a notable observation was that the attenuation ratio between RCC and non-RCC did not differ significantly, whereas the heterogeneity score did. This contrast between the non-significant attenuation ratio and the significant heterogeneity score in renal masses can be attributed to the differing parameters of each variable’s measures. The attenuation ratio from CT scans reflects the average X-ray attenuation of tumor tissue, thereby indicating the lesion's overall attenuation level. However, due to the high structural heterogeneity of renal tumors, this average value may be skewed by a mixed composition of solid tissue, necrosis, bleeding, resulting in subtle distinctions between tumor subtypes that are statistically insignificant [32]. For instance, both papillary or chromophobe RCC and TCC can present similar density values [5,21]. In contrast, the heterogeneity score serves as a more comprehensive metric for evaluating spatial and textural variations in tumor imaging. It encompasses intensity distribution patterns, texture, and the presence of areas of necrosis, fibrosis, and microvascularization within the tumor. This score effectively captures the internal complexity of tumors, demonstrating heightened sensitivity to histopathological heterogeneity associated with tumor size, aggressiveness, and composition [32]. Consequently, while the attenuation ratio may not reveal significant differences, the heterogeneity score is likely to reflect more intricate microstructural changes in tumors, thus offering improved diagnostic and prognostic value in the assessment of renal masses. These findings highlight the critical role of image heterogeneity parameters in renal tumor radiology research, providing insights beyond those derived from mean attenuation values alone. This underscores the need to advance sophisticated radiomic imaging techniques for the detection, characterization, and prognosis of renal tumors [32]. This study offers valuable insights into the heterogeneity score of preoperative CT scans for the identification of malignant renal masses. However, several limitations warrant attention. Notably, the evaluation is subject to greater subjectivity, which can lead to inconsistencies, particularly when masses are relatively homogeneous. Additionally, the study's retrospective design resulted in a limited sample size due to reliance on complete documentation. Excluding numerous cases that did not meet completeness criteria may introduce selection bias and limit the generalizability of the findings. Furthermore, the small sample size could impact statistical power, potentially obscuring clinically relevant differences among some parameters. Thus, we recommend prospective studies with larger sample sizes to yield more robust, representative outcomes. It is also important to note that this study was conducted at a referral hospital, meaning that the patient cohort consisted of individuals requiring further treatment after initial management at their primary hospitals.

## Conclusion

This study suggests that mass heterogeneity may serve as a more reliable diagnostic and prognostic parameter than the attenuation ratio in differentiating malignant renal mass subtypes. Accordingly, heterogeneity assessment on preoperative CT may represent a valuable radiologic biomarker for subtype differentiation and may assist in clinical decision-making and therapeutic planning. Further studies with larger sample sizes are warranted to validate these findings.

### What is known about this topic

- The ratio of Hounsfield Unit (HU) values between a renal mass and the renal cortex (attenuation ratio) is a known and utilized quantitative parameter in CT imaging for initial mass characterization;
- Visual assessment of a tumor's internal texture (heterogeneity) on CT scans is recognized as a qualitative radiological feature often associated with more complex, aggressive, or advanced pathological subtypes.

### What this study adds

- This study provides comparative evidence that visual assessment of mass heterogeneity is a more reliable computed tomography biomarker for differentiating RCC from non-RCC malignant masses than the quantitative attenuation ratio, which showed no significant discriminatory value;
- This study demonstrates that a simple, qualitative 5-point Likert scale for scoring heterogeneity on routine corticomedullary phase computed tomography scans can reflect underlying tumor complexity and aggressiveness, offering a readily applicable tool for radiologists;
- The findings suggest a shift in diagnostic focus from purely attenuation-based metrics to texture analysis, proposing heterogeneity as a key preoperative radiological biomarker to aid in subtype differentiation and clinical planning.
